# The influence of working conditions on health satisfaction, physical and mental health: testing the effort-reward imbalance (ERI) model and its moderation with over-commitment using a representative sample of German employees (GSOEP)

**DOI:** 10.1186/s12889-019-7187-1

**Published:** 2019-07-29

**Authors:** Carolin Kunz

**Affiliations:** Faculty of Sociology, Bielefeld University, Universitätsstraße 25, 33615 Bielefeld, Germany

**Keywords:** Effort-reward imbalance, SF, 12v2™, German Socio–Economic Panel (GSOEP), Work stress model, Health satisfaction, Interaction effects, Moderation

## Abstract

**Background:**

The effort-reward imbalance (ERI) model is well-established in explaining work-related stress and health differences. A lack of reciprocity between efforts and rewards at the workplace is central to the theory. The third component (over-commitment) was defined to be a moderator of high-cost/low gain-working conditions increasing the risk of ill-health. Although the theory has been widely supported empirically, all underlying hypotheses have not been sufficiently tested. This article examines whether the strength of the effect of the effort-reward imbalance ratio on health indicators is bigger than the effects of efforts and rewards individually. Another research gap on the interaction with over-commitment is addressed and health measures are compared.

**Methods:**

This study applied the effort-reward imbalance model on health satisfaction and the SF-12v2™ indicators physical health composite score (PCS) and mental health composite score (MCS) within a representative sample of German employees. After confirmatory factor analyses of the items of the components effort, reward and over-commitment were applied, multiple linear regression models and interaction effects were calculated for more than 7000 respondents within the German Socio-Economic Panel (GSOEP) study.

**Results:**

Against the model’s hypothesis, effort and especially reward had a stronger effect on health satisfaction and mental health individually than the effort-reward imbalance ratio. Over-commitment exerted a negative influence on health indicators and its interaction with the effort-reward imbalance ratio intensified this effect significantly for mental health. Overall, the best model fit was reached for mental health, which is in line with the model’s stress theory foundation.

**Conclusions:**

Although the ERI model has been applied for more than 20 years, theoretical and methodological demands can no longer be neglected. This article contributes to the revision of the effort-reward imbalance (ERI) model and demonstrates possible starting points for prevention programs focusing on rewards.

## Background

Previous research on the explanation of health differences has paid much attention to working conditions as one of the most important determinants of health. In order to meet future demographic challenges and sustain employability, it is even more necessary to preserve employees’ physical well-being and mental health. While the negative effect of physically strenuous work on employee health is readily apparent, the identification of primarily mentally demanding workplaces is more complex and requires a theoretical foundation in particular.

In the 1980s and ‘90s, the “effort-reward imbalance” (ERI) model was introduced to explain the impact of working conditions on employee health in a globalized economy whereas other theories, e.g., the “demand-control” model, focused on industrial societies at that time [1]. The theoretical model connects job strains with rewards and includes the personal characteristic “over-commitment” [2]. The influence of these components and their interplay were formulated in three main hypotheses, which have so far not been sufficiently tested. Especially the interaction of over-commitment and effort-reward imbalance has often been neglected [3, 4]. The clarification of these essential assumptions is crucial for refining the theory and also for identifying possibilities to preserve and improve employees’ physical and mental well-being. In addition, this paper draws a direct comparison between different health indicators and examines whether health satisfaction, physical health or mental health was predicted the best by the ERI model. In this way, it could be examined if health satisfaction – measured by only one question – might also be an appropriate substitute for extensively collected health items.

Although the ERI model is rooted in medical sociology, it is based on theories on social exchange and stress [5]. Reciprocity – being the core of the theory – is considered as “the vital principle of society” [6]. This internalized moral norm contributes to the stability of societies. In detail, “certain actions and obligations [are defined] as repayments for benefits received” [7]. Siegrist [2] transferred this principle to a vocational setting. Non-reciprocity of efforts and rewards in a working environment may lead to strong negative emotions and distress because an imbalance violates a contractually fixed exchange relationship [5]. At this point, it has to be distinguished between the everyday use of the terms stress, which usually refers to pressure of time, and distress. Siegrist’s definition is based on Selye [8] but goes one step further: Situations are perceived as stressful when routines are interrupted by threats and challenges that force individuals to take action in order to cope with the situation [2, 5]. Consequently, challenging situations turn out to cause distress if coping fails [9]. The ERI model also highlights the interindividual differences in handling distress, which emphasizes the importance of the subjective perception of working conditions for the operationalization in empirical studies [5, 9].

In case of negative emotions caused by an imbalance of costs and gains, the two stress axes and, as a result, the autonomic nervous system are activated, which can lead to physical and psychological diseases when chronification occurs [2, 5, 9]. Permanent activation and the inability to return to normal conditions are referred to as “allostatic load” [10]. Due to a chronic false regulation, recovery is hindered [11], which in turn increases the risk of, e.g., coronary heart diseases, depression, diabetes mellitus, nutritional disturbance or addictions [12, 13].

**Fig. 1:**
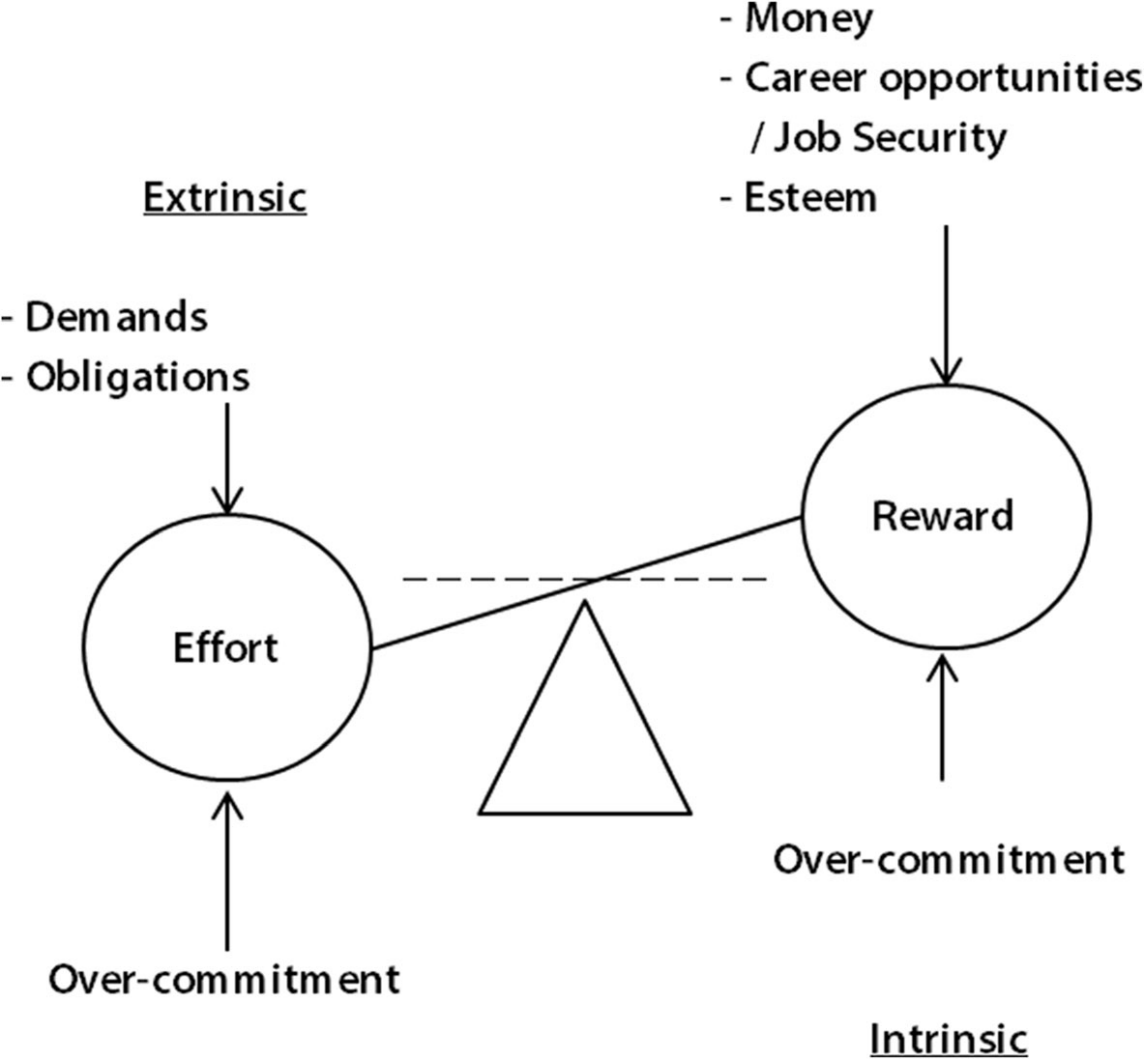
Effort-reward imbalance (ERI) model [5]

The ERI model consists of three components: effort, reward and over-commitment as shown in Fig. 1. Efforts are represented by demands and duties like high workload, frequent interruptions or time pressure whereas salary, esteem, job security and career opportunities are forms of occupational rewards. An imbalance between costs and gains would mean a violation of the norm of reciprocity and – in the long run – may affect health via strain reactions. In order to identify non-reciprocity, scales were developed to collect data on working conditions and hence to obtain a ratio that represents the imbalance [2].

Furthermore, the intrinsic component over-commitment was added to Siegrist’s main idea of hig-cost/low gain-working conditions. It is defined to be “a set of attitudes, behaviours and emotions reflecting excessive striving in combination with a strong desire of being approved and esteemed” [14]. This personal disposition arose out of Type A-behavior and is characterized by strong ambitions combined with a high need for approval and esteem [14]. As displayed in Fig. 1, over-commitment influences the perception of efforts and rewards. Due to an underestimation of challenging tasks and an overestimation of their own skills, overcommitted employees may exaggerate their efforts and tend to need higher rewards which may lead to exhaustion in the long run [2].

According to the expectancy-value theory of motivation [5], individuals should strive for a reduction of the imbalance by lowering their efforts or quitting. Siegrist answered this contradiction of rational choice with three scenarios. An imbalance is maintained when (1) individuals do not have any other or fewer opportunities on the labor market; (2) individuals accept non-reciprocity for strategic career reasons, e.g., in order to be promoted; or (3) individuals are overcommitted [2, 15]. Therefore, over-commitment plays a special role in the ERI model.

Siegrist [1] summarized the model assumptions in the following hypotheses, which will be tested empirically in this article:

1. "Each component of the model, defined by the scales ‘effort’, ‘reward’, and ‘over-commitment’, exerts separate effects on the health outcome under study. In general, these effects reflect a dose-response relationship.
2. The size of effect on health produced by a combined measure quantifying the imbalance between high effort and low reward exceeds the size of effect on health produced by each single component (e.g. as demonstrated by the individually assessed ‘effort/reward ratio’).
3. The personal coping pattern ‘over-commitment’ moderates the effect size of effort-reward imbalance on health (interaction term). Among people scoring high on over-commitment this effect is significantly stronger than among people scoring low on this pattern of coping." [1]

These theoretical arguments can be tested statistically but especially the interaction effect has not been tested in many studies even though moderation is part of Siegrist’s main hypotheses [3, 4]. Accordingly, a high level of over-commitment intensifies the negative impact of effort-reward imbalance on health indicators [3]. Past research highlighted the necessity to test the third model hypothesis containing the moderating role of over-commitment. However, a review of 45 empirical studies on the ERI model revealed that a complete test was only carried out in 12 of those studies. The majority found no significant effect for the interaction term [4]. The in-depth testing of the theory in this paper adds value to the advancement of the theory, as well as it could identify possible starting points for improving employee health: In order to preserve their employability, highly overcommitted employees could be supported in handling actual efforts and perceiving rewards more realistically.

The first and, to some extent, second hypotheses have been confirmed with different dependent variables: At the beginning of the research on the ERI model, surveys focused mainly on cardiovascular diseases [2, 4] but an increasing number of studies have proved the impact of effort-reward imbalance on, e.g., biomedical parameters [2, 5], self-reported health [16], major depression [13, 17], addictions [18] or insomnia [2, 13]. In most of the studies, a strong, negative effect of effort-reward imbalance was found on health indicators whereas over-commitment was often neglected [4]. This paper aims to close the research gap and includes over-commitment in the empirical analysis as it is actually outlined in the third hypothesis.

In contrast to previous research, different variations of the ERI model need to be compared by calculating multiple linear regressions and interaction effects. Hence, the aim of this article is to clarify the relationship between effort, reward and over-commitment in order to explain their impact on health. After presenting the underlying data and variables, I will compare different model assumptions based on the German Socio-Economic Panel (GSOEP) survey. Implications for further research will be given in the discussion.

## Methods

### Study sample

The GSOEP is a representative longitudinal household panel study conducted by the German Institute for Economic Research (Deutsches Institut für Wirtschaftsforschung e.V.). More than 22,000 individuals in about 12,000 households are interviewed annually. The GSOEP was begun in West Germany in 1984 and in East Germany in 1990. Focusing on the “analysis of the life course and well-being” [19], it covers a wide range of multidisciplinary topics like health indicators, job-related characteristics or sociodemographic items [19].

The relevant variables for the ERI model are collected every five years starting in 2006 [20]. However, the operationalization of effort and reward has changed over time: In 2011, employees were first asked about the occurrence and subsequently about the level of distress of efforts and rewards. In this study, I used data from 2016 where respondents were only asked to rate efforts and rewards on a 4-point scale from (1) “strongly disagree” to (4) “strongly agree” in a one-step procedure. These two measurements are not comparable [21] and therefore longitudinal analyses were unfortunately impossible. Thereby, causality could not be tested empirically but can be assumed theoretically.

In order to obtain a more homogeneous sampling unit, I excluded individuals who were older than 64 years, as well as handicapped, unemployed, self-employed, retired, family workers, in military or civilian service, in vocational training or internship, in maternity or parental leave. I also restricted the analysis to employees working 30 or more hours per week. Due to the exclusion of employees with less than 30 working hours, the majority of the respondents were male (60%). The mean age was round about 43.2 years and 75% had an open-ended full-time contract.

### Measurements and statistical analyses

In this article, three different indicators for measuring health are used and compared: The physical health composite score (PCS), mental health composite score (MCS), and employee’s health satisfaction. Especially the latter is a global measure to assess health in an economic way but, to the best of my knowledge, this item has not been used frequently in analyses with the ERI model. Therefore, more insight on the influence of working conditions on health satisfaction will be given in this paper by also comparing this measure to the alternative health indicators PCS and MCS.

PCS and MCS are components of the widely approved SF-12v2™ questionnaire which were included in the GSOEP data [22]. The SF-12v2™ contains 12 items, e.g., health status, health impairments, and limitations (as listed in Table 3 in the Appendix), and is a shorter version of the SF-36v2™. These variables were grouped into eight subscales and in turn categorized in the subordinate dimensions “physical health” (PCS) and “mental health” (MCS) [23]. Besides a slightly different wording, the layout and order of the items differed between the GSOEP and the original questionnaire [22]. The German Institute for Economic Research provided the indices on the subscales PCS and MCS. Lower values represented a worse health status. The 2016 GSOEP survey provided information on both indicators for 8627 respondents. Arithmetic mean values were very similar (mean_pcs_ = 52.31; mean_mcs_ = 52.17) and their range differed slightly (Min_PCS_ = 13.98; Max_PCS_ = 73.06; Min_MCS_ = 7.46; Max_MCS_ = 73.14). The mean values were presumably higher than in 2004 [22] because respondents older than 64 years were excluded. Women had lower values than men, meaning that they reported a worse physical and mental health.

In addition, employees’ health satisfaction (“How satisfied are you with your health?”, which was measured on an 11-point scale from (0) “completely dissatisfied” to (10) “completely satisfied” in the GSOEP survey [24]) was used for a comparative analysis of health indicators. Compared to PCS and MCS, health satisfaction is a parsimonious way of measuring health in surveys. Besides this methodological advantage, respondents could rate their satisfaction with health in regard to one’s expectations, personal aims, or restrictions caused by potential diseases [25]. In contrast to the operationalization of diagnosed diseases, a subjective measure also covers health impairments like undetected or developing diseases. Respondents even have the possibility to weigh their impairments by severity and (future) courses of diseases [26]. In previous research, the subjective health status was found to be a predictor for objective measures of morbidity and even mortality [26, 27]. In the GSOEP survey, 8755 respondents reported their health satisfaction. The overall health satisfaction was relatively high among the respondents (mean = 7.23) whereas women were less satisfied than men (mean_Women_ = 7.11).

In the last decades, the ERI model has been widely tested empirically and refined [21]. Especially the questionnaire was reduced in order to be applied in surveys in different occupational areas [9]. In 2016, effort-reward imbalance was measured by this updated short version of the questionnaire. Effort consisted of three items (“There is often high time pressure due to the large volume of work,” “People often interrupt or bother me while I’m working,” and “My workload has increased steadily over the last two years”) whereas seven items were asked on reward (“The chances of promotion are low where I work,” “My work situation is getting worse or I am expecting it to get worse in the future,” “My own job is at risk,” “I receive the recognition I deserve from my superiors,” “When I consider all my accomplishments and efforts, the recognition I’ve received seems about right to me,” “When I consider all my accomplishments and efforts, my personal chances of career advancement seem about right to me,” and “When I consider all my accomplishments, my pay seems about right to me”) [24]. As mentioned before, effort and reward were rated on a 4-point scale from (1) “strongly disagree” to (4) “strongly agree” like the six items for over-commitment (“I often am already thinking about work-related problems when I wake up,” “When I come home, it is very easy to switch off from thinking about work,” “Those closest to me say I sacrifice myself too much for my career,” “Work seldom lets go of me; it stays in my head all evening,” and “If I put off something that needs to be done that day, I can’t sleep at night”) [24].

Before three sum scores were generated by adding each item for each component as recommended by Siegrist [5], confirmatory factor analyses were calculated in Mplus. The underlying factorial structure of effort, reward and its subcomponents, and over-commitment was not confirmed in the first step (RMSEA = 0.073; CFI = 0.894; TLI = 0.871; SRMR =0.054). One item for over-commitment (“At work, I easily get into time pressure”) was excluded because it was similar to one item for effort (“There is often high time pressure due to the large volume of work”). Respondents might have not distinguished between time pressure arising due to internal and external reasons. The correlation between these two variables for time pressure was found to be strong (Pearson’s r = 0.61, *p* = 0.000). In addition, the eliminated variable for over-commitment differed thematically from the others, which implied sacrifice for the career and a lack of psychological detachment from work. The second confirmatory factor analysis without over-commitment’s variable for time pressure supported these doubts: The fit of the reduced model was acceptable (RMSEA = 0.052; CFI = 0.949; TLI = 0.936; SRMR =0.044), which justified the use of additive indices for effort, reward, and over-commitment without time pressure. The sum scores were mean-centered in order to be able to interpret the intercept properly because the value “0” does actually exist on the scale representing the mean value. Centering is a linear transformation of metric variables, which does not influence the interpretation of the regression coefficients [28]. Mean-centered effort ranged from − 4.80 to 4.20, mean-centered reward from − 12.25 to 8.75, and over-commitment from − 5.37 to 9.63. High positive values for effort indicated higher efforts than the average respondents, whereas high negative values represented lower efforts than the mean value of the interviewed employees. The interpretation of reward and over-commitment was comparable. Another advantage of mean-centering the variables was the greater approximation to the normal distribution, which is a condition for regression analyses [28].

In the next step, a ratio of the original sum scores of effort and reward (not mean-centered) was calculated following this formula:

$$ ERI\  Ratio=\frac{centered\  sum\  score\ effort}{centered\  sum\  score\ reward\ast \frac{number\ of\ items\  on\  effort}{number\ of\ items\  on\  reward}} $$

The variables for efforts were divided by rewards and a correction factor, which adjusted for the different number of items. High scores indicated a high job strain [9]. In order to avoid multicollinearity in the regression models with interaction effects [28], the ERI ratio was also mean-centered and ranged from − 0.77 to 2.98. Higher values indicated a greater deviation from the survey mean of ERI and therefore a stronger violation of the norm of reciprocity. In order to get an overview with descriptive statistics, another variable for ERI with four categories was generated based on the quartiles as recommended [9, 15, 18, 21]. In previous papers suggested classifications of imbalanced jobs by using the cut off-point “1”, which was used in the majority of articles on effort-reward imbalance [4], were revised because the amount of stressful workplaces was overestimated [21]. This is also the case in the here used dataset because the mean value of the original ERI ratio was 1.02.

The preparation of the data set and the multiple linear regression models were conducted with the statistical program Stata 14.2. The models were adjusted for sex, mean-centered age, and the mean-centered amount of education or training in years. As mentioned above, the third hypothesis implied a moderation of over-commitment. Thus, an interaction term between the mean-centered sum score of over-commitment (without the item for time pressure) and the mean-centered metric ERI ratio was generated and added to the regression models.

## Results

At first, a short insight into the relationship between ERI and health is provided. More than 60% of the respondents were in the third and fourth ERI quartile and thus had a higher risk of reduced health according to the ERI model. The proportion of women in the first and second quartile was slightly lower than men.

**Fig. 2:**
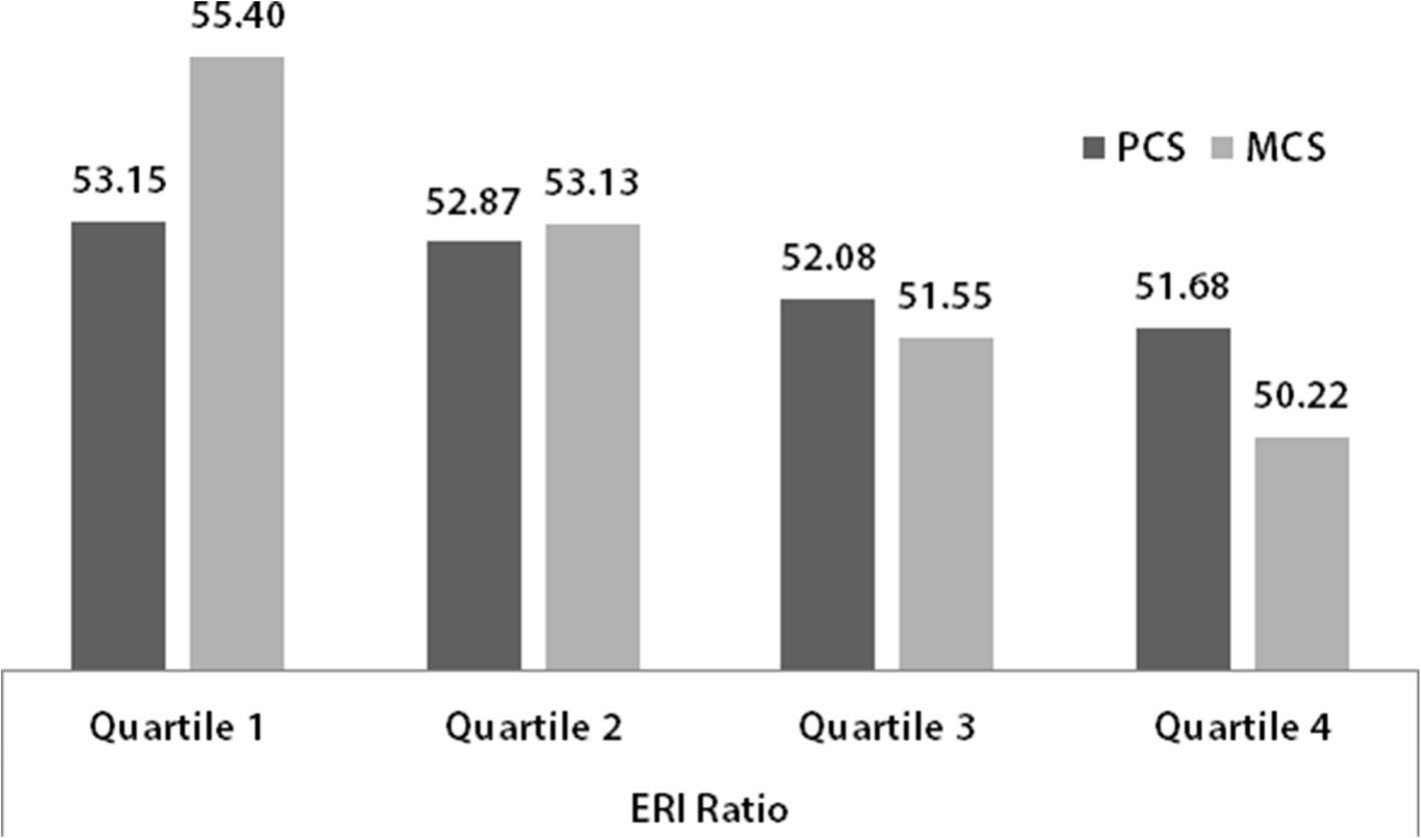
Mean values of PCS and MCS by ERI quartiles (GSOEP, *n* = 8627)

The mean value of health satisfaction differed between the ERI quartiles (Health satisfaction_Quartile 1_ = 7.65; Health satisfaction_Quartile 4_ = 7.01). A higher ERI ratio also went along with worse physical and mental health (Fig. 2). The largest difference between the quartiles was found for MCS. This finding was a first indicator for the strong effect of ERI on mental health, which will be examined in more detail in the multivariable analyses.

The correlation between health indicators and the mean-centered ERI ratio was negative (Pearson’s *r*_Health satisfaction_ = − 0.23; *p* = 0.000; Pearson’s *r*_PCS_ = − 0.15; *p* = 0.000; Pearson’s *r*_MCS_ = − 0.32; *p* = 0.000). Consequently, employees with a higher deviation from the average ERI ratio and therefore more stressful working conditions had a worse health. As already mentioned, the relationship between ERI ratio and mental health was stronger than between ERI ratio and health satisfaction or physical health. This was also the case for the correlation between the mean-centered variable for over-commitment and health indicators (Pearson’s *r*_Health satisfaction_ = − 0.18; *p* = 0.000; Pearson’s *r*_PCS_ = − 0.09; *p* = 0.000; Pearson’s *r*_MCS_ = − 0.34; *p* = 0.000). The high correlation between the mean-centered variable for over-commitment and the mean-centered ERI ratio (Pearson’s *r* = 0.40; *p* = 0.000) was remarkable as well as between the mean-centered variable for over-commitment and the mean-centered variable for effort (Pearson’s *r* = 0.47; *p* = 0.000). This finding could indicate that the components of the ERI model are strongly interrelated. This, in turn, could have led to an underestimation of the effects in the subsequent regression analyses due to multicollinearity.

In order to test Siegrist’s hypotheses [1], multiple regression models were estimated for health satisfaction, physical and mental health. First, I included the independent variables mean-centered ERI ratio, effort, reward, and the control variables (Table 1). According to the first hypothesis, the ERI ratio should have a stronger effect on health than its components individually. This was only the case for physical health because the standardized regression coefficient of the mean-centered ERI ratio differed more from 0 than those of effort and reward. In the regression models for health satisfaction and MCS, reward had the strongest influence. This led to a rejection of Siegrist’s first hypothesis. In contrast, the influence of working conditions on physical health was found to be smaller. Less than 10% of the variance was explained.

**Table 1:** Results of linear regression models with health indicators (GSOEP)

	Health satisfaction				Physical health composite score (PCS)				Mental health composite score (MCS)			
	Coef.	Stand. Coef.	Coef.	Stand. Coef.	Coef.	Stand. Coef.	Coef.	Stand. Coef.	Coef.	Stand. Coef.	Coef.	Stand. Coef.
ERI ratio (centered)	-.32*	-.08	-.70**	-.17	-1.97**	-.12	-1.84**	-.11	-1.79**	-.09	-4.18**	-.21
Effort (centered)	-.04*	-.05	-	-	.09	.03	-	-	-.51**	-.13	-	-
Reward (centered)	.06**	.14	-	-	.09*	.04	-	-	.39**	.18	-	-
OC (centered)	-	-	-.06**	.11	-	-	-.11**	-.05	-	-	-.70**	-.26
Age (centered)	-.03**	-.18	-.03**	-.18	-.17**	-.22	-.17**	-.22	.05**	.06	.06**	.07
Sex (Ref. male)	-.14**	-.04	-.12**	-.03	-.73**	-.05	-.71**	-.04	-1.83**	-.10	-1.55**	-.09
Education or training in years (centered)	.05**	-.07	.06**	.09	.46**	.16	.50**	-.17	.01	-.00	.11**	.04
Cons.	7.21**		7.20**		52.31**		52.30**		52.71**		52.58**	
Adjusted R²	.1002**		.1029**		.0988**		.0989**		.1204**		.1689**	
n	7.176		7.115		7.087		7.039		7.087		7.039	

Significance: **= *p* ≤ .01; *= *p* ≤ .05; *Coef.* Unstandardized coefficients, *Stand. Coef*: Standardized Coefficients

In the next step, the mean-centered variable for over-commitment was included and the mean-centered items for effort and reward were excluded from the regression models in order to test the second hypothesis. Compared to the first models, the explanatory power was basically the same except for the MCS model: Nearly 17% of MCS’s variance was explained by the mean-centered variables for ERI, over-commitment and control variables. Over-commitment exerted a significant negative influence on health indicators, which is in line with the second hypothesis: The higher the over-commitment value, the worse is employees’ health. Besides, over-commitment had the strongest effect on MCS.

According to the third hypothesis, employees with a high ERI ratio and high over-commitment values are at the highest risk for health impairments. This assumption was modeled with regression models including an interaction effect (Table 2).

**Table 2:** Moderation of over-commitment in regression models (GSOEP)

	Health satisfaction		Physical health composite score (PCS)		Mental health composite score (MCS)	
	Coef.	Stand. Coef.	Coef.	Stand. Coef.	Coef.	Stand. Coef.
ERI ratio (centered)*OC (centered)	-.01	-.01	.06	.01	-.17**	-.04
ERI ratio (centered)	-.70**	-.17	-1.88**	-.11	-4.03**	-.21
OC (centered)	-.06**	-.10	-.11**	-.05	-.69**	-.26
Age (centered)	-.03**	-.18	-.17**	-.22	.06**	.07
Sex (Ref. male)	-.12**	-.03	-.72**	-.05	-1.54**	-.08
Education or training in years (centered)	.06**	.09	.50**	.18	.11**	.03
Cons.	7.21**		52.27**		52.69**	
Adjusted R²	.1028**		.0989**		.1701**	
n	7.115		7.039		7.039	

Significance: **= p ≤ .01; *= p ≤ .05

Coef.: Unstandardized coefficients; Stand. Coef: Standardized Coefficients

**Fig. 3:**
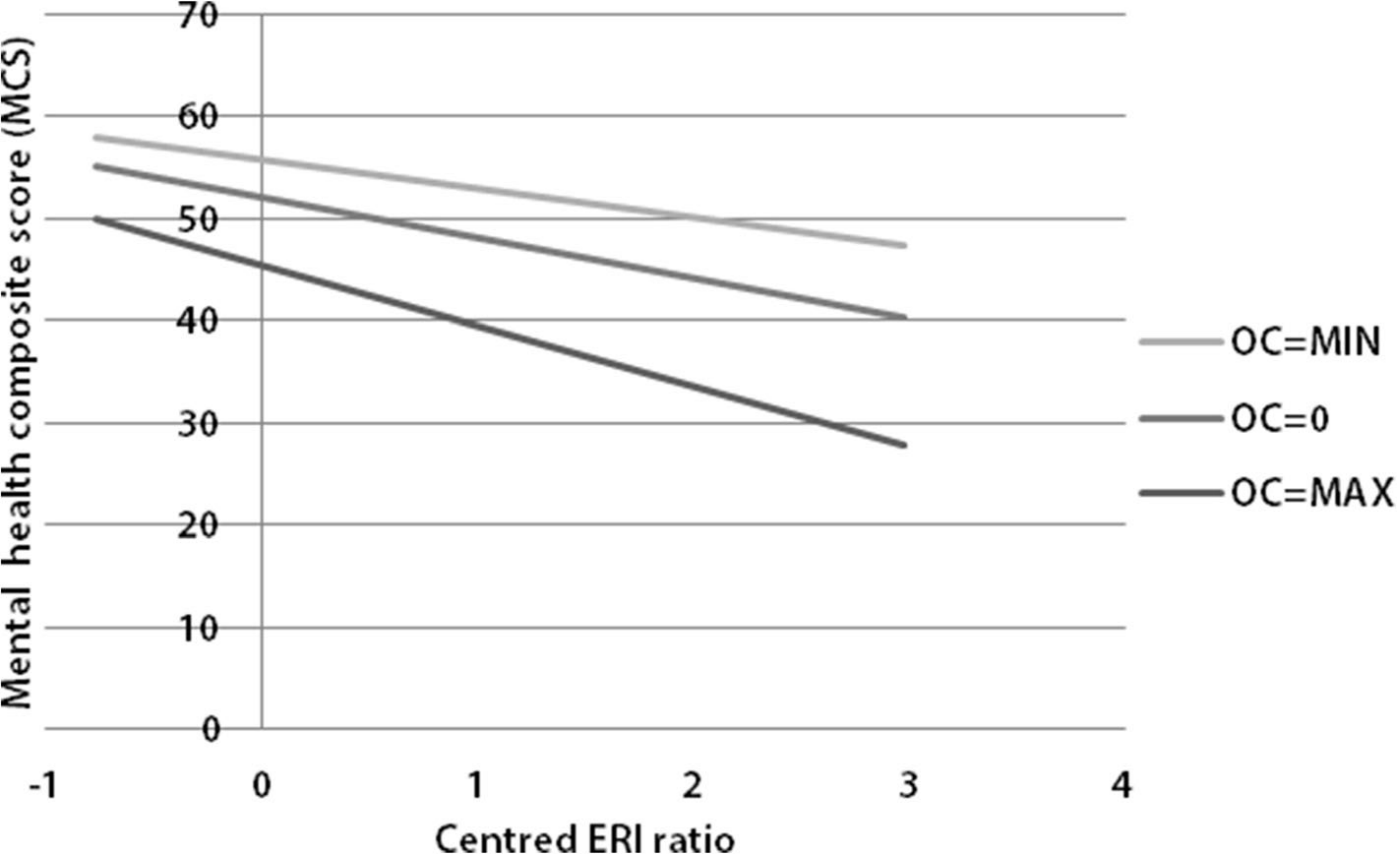
Moderation of mean-centered over-commitment and mean-centered ERI ratio on the mental health composite score (MCS) (GSOEP, *n* = 7248, without control variables)

Siegrist’s interaction hypothesis was not supported for health satisfaction and physical health because it was not valid for the population. Besides these insignificant regression coefficients, the model fit was not better than in the models without interaction effects (see R^2^ values). Only in the mental health model, the interaction effect between mean-centered ERI ratio and mean-centered over-commitment exerted a significant, negative influence on MCS. Therefore, being overcommitted intensified the pathogenic influence of occupational non-reciprocity. Figure 3 illustrates the interplay of the variables in the model, where the control variables were left out for reasons of simplicity because they only exerted a very small influence on mental health. Employees with the lowest values for the mean-centered ERI ratio in combination with the minimum of mean-centered over-commitment values had the highest mental health values. Respondents with the maximum mean-centered ERI ratio and the highest mean-centered over-commitment values were observed to have poorer mental health.

## Discussion

During the long history of the ERI model, the relationship between non-reciprocity and health outcomes has been confirmed innumerable times [3, 4] although Siegrist’s main hypotheses have not been sufficiently tested. With data of the GSOEP study, I was able to show that the ERI ratio has a negative effect on health indicators but the strength of its influence (standardized coefficients) was not bigger than the individual effects of effort and reward on health satisfaction and mental health. In these models, Siegrist’s first hypothesis was not supported by representative data of more than 7000 respondents. Another reason for the individual use of the single components is the fact that probably too much complexity is lost by using an ERI ratio, because reward exerted the strongest influence on health satisfaction and mental health. With this knowledge, it might be easier for experts and practitioners to develop intervention programs in order to preserve employee health and create healthy working conditions. Workplace health promotion could include leadership development towards an honoring working environment and a sensitization with respect to mental health problems caused by adverse working conditions. In this context, it might also be more comprehensible and easier for companies to develop a culture of recognition than to understand the image of an effort-reward imbalance.

In line with Siegrist’s second hypothesis, over-commitment exerted a negative influence on all of the three health indicators. According to the models, the inability to switch off from thinking about work or having work-related problems after waking up led to worse mental health. By including over-commitment in the mental health model, the R^2^ value rose from 12.04 to 16.89. This also indicates that the ERI model with over-commitment held true rather for mental health than for physical health. These results support the ERI model’s basis being stress theory: Working conditions might have caused negative emotions, which may lead to an activation of the two stress axes and the autonomic nervous system, causing physical diseases in the long run [2, 5, 9]. This could be the reason for the comparable low percentage of explained variance of physical health: The rather weak and partially insignificant relationship between variables for ERI and physical health might have been due to the study design because pathogenic working conditions may exert a time-delayed influence on physical health. Due to the use of cross-sectional data, assumptions on causality could only be drawn theoretically but the detected relations between the variables at least indicated a cause and effect relationship since correlation is one condition for causality. In any case, future research should be based on longitudinal data. The data used in this study, however, only took one time point into consideration.

In addition, the use of the SF-12v2™ questionnaire might have also led to a weaker relationship between the ERI variables and physical health than in previous studies. Having problems climbing stairs or lifting something heavy could occur more frequently to older respondents, which was underlined by the high standardized regression coefficient of age. Consequently, these severe health impairments might be more a matter of age or additionally biased: Respondents suffering from serious limitations by their health might have already been retired and therefore excluded from the dataset. Overall, it was widely unexplored whether health satisfaction or the SF-12v2™ questionnaire was similarly influenced by ERI variables. Thus, this paper might contribute to the discussion on the use of global subjective health measures such as health satisfaction. As a whole, the ERI model contributed less to the explanation of PCS than to health satisfaction or MCS, as displayed by the model fit indices. Presumably, respondents understood health satisfaction not only as physical but also mental health. Therefore, future research could open up to the use of the indicator health satisfaction.

Unlike the majority of studies on the ERI model [3], this paper investigated the interaction of the ERI variables and over-commitment. The third hypothesis was partially supported: Over-commitment only intensified the effect of the ERI ratio significantly on mental health. Being strongly over-committed and having a lack of reciprocity led to worse health than reciprocal working conditions and low levels of over-commitment. Another methodological strength of this analysis is that it tried to respect the distributional assumptions by mean-centering the variables for the ERI model. This is a condition for conducting linear regression models but was often neglected in past research. A limitation of this study is that the dependent variables in this paper were only roughly normally distributed. Future analyses should include the Satorra-Bentler-correction factor [29] in order to handle non-normal variables. In addition, future studies should take methods of empirical social research more into consideration and the choice of control variables needs to be well thought out: Particularly worth mentioning is also the finding that effort became insignificant in the PCS model when the item for the amount of education or training was introduced. Presumably, efforts differed by educational background, so that the effect of efforts would have been overestimated if the models were not controlled for education. Consequently, the use of reasonable control variables is indispensable.

Finally, this paper highlights the need for revising the concept of the ERI components and their interplay because the here mentioned confirmatory factor analyses revealed that time pressure (“At work, I easily get into time pressure,” which belongs to over-commitment) had to be excluded. Although this variable was excluded for over-commitment, stepwise regression models revealed that the effect size of the mean-centered ERI ratio decreased strongly when the mean-centered variable for over-commitment was introduced (results not displayed). This indication for their statistical dependency suggests that a mediation analysis via path or structural equation models is needed. Nevertheless, this statistical hint needs to be based on theoretical considerations. Consequently, a closer look should be taken at the components and especially over-commitment in particular. Over the years, its item set was reduced for parsimony reasons but does it still represent the main idea of “a set of attitudes, behaviors and emotions that reflect excessive striving in combination with a strong desire of being approved and esteemed” [14]? Du Prel and colleagues [30] already doubted the role of over-commitment as a trait influencing the perception of efforts and rewards. In addition, four of the here used items for over-commitment represent the inability to detach from work. Thus, the role of over-commitment in the model has to be reconceptualized from influencing the perception of efforts and rewards toward failed detachment being a consequence of high efforts and low rewards, as Sonnentag [31, 32] discovered in longitudinal studies. For these reasons, the model comparisons offered one step towards explaining work-related health differences but future research should focus on the clarification of over-commitment in order to improve the ERI model.

## Conclusions

In summary, effort and especially rewards exerted a stronger negative effect on health satisfaction and mental health than the effort-reward imbalance ratio. Over-commitment had a negative influence on the here used health indicators. Compared to the mental health model, a lower amount of physical health’s variance was explained by working conditions, which might be due to the severity of the indicator or the cross-sectional design of the analyses. Working conditions might impair employees’ health in the long run via a reduction of mental health. Especially mental health was reduced when a combination of high values of the effort-reward imbalance ratio and over-commitment were observed.

## Data Availability

The data that support the findings of this study are available from Deutsches Institut für Wirtschaftsforschung e.V. but restrictions apply to the availability of these data, which were used under license for the current study, and so are not publicly available. Data are however available from the authors upon reasonable request and with permission of Deutsches Institut für Wirtschaftsforschung e.V.. Please find the questionnaires here: https://www.diw.de/documents/publikationen/73/diw_01.c.571045.de/diw_ssp0345.pdf

## Appendix

**Table 3:** GSOEP SF–12™ indicators

Subscale	Item	Dimen-sion
General Health	How would you describe your current health?	PCS
Physical Fitness	When you have to climb several flights of stairs on foot, does your health limit you greatly, somewhat, or not at all?	PCS
Physical Fitness	And what about other demanding everyday activities, such as when you have to lift something heavy or do something requiring physical mobility: Does your health limit you greatly, somewhat, or not at all?	PCS
Mental Health	During the last four weeks, how often did you feel down and gloomy?	MCS
Mental Health	During the last four weeks, how often did you feel calm and relaxed?	MCS
Vitality	During the last four weeks, how often did you feel energetic?	MCS
Bodily Pain	During the last four weeks, how often did you have severe physical pain?	PCS
Role Physical	During the last four weeks, how often did you feel that due to physical health problems you achieved less than you wanted to at work or in everyday activities?	PCS
Role Physical	During the last four weeks, how often did you feel that due to physical health problems you were limited in some way at work or in everyday activities?	PCS
Role Emotional	During the last four weeks, how often did you feel that due to mental health or emotional problems you achieved less than you wanted to at work or in everyday activities?	MCS
Role Emotional	During the last four weeks, how often did you feel that due to mental health or emotional problems you carried out your work or everyday tasks less thoroughly than usual?	MCS
Social Functioning	During the last four weeks, how often did you feel that due to physical or mental health problems you were limited socially, that is, in contact with friends, acquaintances, or relatives?	MCS

*PCS* physical health composite score, *MCS* mental health composite score

## Abbreviations

ERI: Effort-reward imbalance
GSOEP: German Socio-Economic Panel
MAX: Maximum
MCS: Mental health composite score
MIN: Minimum
OC: Over-commitment
PCS: Physical health composite score
SF-12v2™ questionnaire: Short-Form 12-Item Health Survey
